# Prognostic significance of 18F-Fluorodeoxyglucose positron-emission tomography parameters in patients with biliary tract cancers: a meta-analysis

**DOI:** 10.1186/s12880-023-01182-4

**Published:** 2024-01-02

**Authors:** Xia Zheng, Yue Shi, Delida Kulabieke, Zihao Wang, Ying Cheng, Jun Qian

**Affiliations:** 1 Oncology Department, Affiliated Hospital of Nanjing University of Chinese Medicine, Jiangsu Province, No.155 Hanzhong Avenue, Nanjing, 210000 China; 2 Dermatology Department, Affiliated Hospital of Nanjing University of Chinese Medicine, Jiangsu Province, No.155 Hanzhong Avenue, Nanjing, 210000 China

**Keywords:** 18F-Fluorodeoxyglucose positron-emission tomography, Biliary tract cancer, Prognosis, Meta-analysis

## Abstract

**Background and objective:**

Numerous previous studies have assessed the prognostic role of 18F-fluorodeoxyglucose positron-emission tomography (18F FDG PET) in patients with biliary tract cancer (BTC), but those results were inconsistent. The present study aims to determine the predictive value of 18F FDG PET in BTC patients via a meta-analysis.

**Methods:**

The underlying studies related to 18F FDG PET and BTC patients` outcomes were searched and identified in the online databases. The interested parameters include total lesion glycolysis (TLG), metabolic tumor volume (MTV), primary tumor and metastatic lymph node (LN) maximum standardized uptake value (SUVmax), as well as change of SUVmax (ΔSUVmax) during treatment. Overall survival (OS), disease-free survival (DFS), and progression-free survival (PFS) were considered as the primary endpoints. Hazard ratio (HR) and corresponding 95% confidence intervals (CIs) were defined as the effective measure and calculated by a pooled analysis. Publication bias was assessed by funnel plot, Bagg’s and Egger’s tests.

**Results:**

Totally, 23 studies involving 1478 patients were included in the present meta-analysis. After a pooled analysis, it revealed that a high SUVmax was significantly associated with a poor OS (HR:2.07, 95%CI: 1.74–2.46, *P* = 0.000) and DFS (HR: 2.28, 95%CI: 1.53–3.41, *P* = 0.000). In addition, an increased TLG level contributed to a shorter OS (HR:1.91, 95%CI: 1.26–2.90, *P* = 0.002) and DFS (HR: 4.34, 95%CI: 1.42–13.27, *P* = 0.01). Moreover, we confirmed that an elevated MTV was significantly associated with increased mortality (HR:2.04, 95%CI:1.26–3.31, *P* = 0.004) and disease relapse (HR: 3.88, 95%CI:1.25–12.09, *P* = 0.019) risks. Besides, the present study uncovered that increased ΔSUVmax could predict poor OS (HR:1.26, 95%CI:1.06–1.50, *P* = 0.008) instead of PFS (HR: 1.96, 95%CI: 0.82–4.72, *P* = 0.280). Lastly, we found that LN SUVmax did not link to OS (HR: 1.49, 95%CI: 0.83–2.68, *P* = 0.178). No obvious publication bias was detected in the present study.

**Conclusion:**

18F FDG PET parameters, including SUVmax, TLG, MTV, and ΔSUVmax, could be applied as convenient and reliable factors for predicting BTC patients` outcomes.

**Supplementary Information:**

The online version contains supplementary material available at 10.1186/s12880-023-01182-4.

## Introduction

As a highly heterogeneous disease, biliary tract cancer (BTC), including intrahepatic, perihilar, and distal cholangiocarcinoma, as well as gallbladder and ampulla cancer, is a low-incidence but fatal neoplasm with poor prognosis [1]. The global morbidity of intrahepatic cholangiocarcinoma (ICC) is rising gradually, especially in low-income countries [2]. Despite the application and development of several examination methods and treatment options, patients` overall survival (OS) remains limited [3]. Surgical resection is the primary curative option for early-stage BTC patients. Unfortunately, most patients develop locally advanced or metastatic disease when diagnosed due to a lack of particular symptoms in the early stage. Their survival is restricted, although palliative chemotherapy has been recommended. In addition, most postoperative patients would suffer from disease relapse, which limited their 5-year OS rate to approximately 20–60% [4]. So far, the recurrent and mortality risks of BTC remain less understood. It is essential to explore the underlying prognostic factors to identify the high-risk population in order to achieve precision management.

As an imaging technique based on glucose metabolism to assess a variety of physiological and disease processes, 18F-fluorodeoxyglucose positron-emission tomography (18F FDG PET) has been defined and applied in staging and managing multiple cancers, including BTC. Interestingly, numerous previous meta-analyses determined that some parameters of 18F FDG PET could be utilized as prognostic factors in patients with gastric cancer [5], pancreatic cancer [6], and lung cancer [7] instead of BTC. Meanwhile, the predictive significance of 18F FDG PET parameters has been assessed in BTC by previous clinical studies. However, these results were inconsistent due to different sample sizes and study designs. For example, the cohort studies conducted by Seo et al. [8] and Yho et al. [9] demonstrated that the maximum standardized uptake value (SUVmax) of primary tumor mass was an independent predictor for disease-free survival (DFS) and OS. By contrast, some investigators demonstrated that SUVmax did not contribut to BTC patients` outcomes [10, 11]. Therefore, we performed the present meta-analysis to re-assess the prognostic value of multiple 18F FDG PET parameters, including total lesion glycolysis (TLG), metabolic tumor volume (MTV), primary tumor, and metastatic lymph nodes (LN) SUVmax, as well as change of SUVmax (ΔSUVmax) during treatment in patients with BTC.

## Methods

### Search strategy

Published studies potentially related to BTC and 18F FDG PET were searched from the PubMed, Embase, Cochrane Library, and Web of Science databases in August 2022. The keywords “biliary tract cancer,” “cholangiocarcinoma,” “positron-emission tomography,” and “prognosis,” as well as related abbreviations, were used for the screening and identification of candidate studies to be included in the meta-analysis. Multiple synonyms were also utilized.

### Inclusion and exclusion criteria

Eligible studies were identified using the following criteria: (1) studies addressing the relationship between the outcomes of patients with BTC and metabolic parameters of 18F FDG PET, (2) diagnosis of all BTC participants by pathological examination. (3) reported in English.

The exclusion criteria for this meta-analysis were: (1) other types of articles (i.e., reviews, conference abstracts, case reports, or comments); (2) in vivo or in vitro research studies; (3) lack of data on DFS, PFS or OS; (4) lack of hazard ratios (HRs) and 95% confidence intervals (CIs) as practical measurements; (5) involving other pathological types of cancer patients.

### Data management and outcome assessment

According to the above criteria, two investigators independently screened and reviewed available publications through abstract and full-text reading. If there was any disagreement between them, a consensus was reached through discussion with a senior investigator. We collected and defined the HRs and 95% CIs of OS and DFS as the effective measurements. The HRs and 95% CIs calculated by multivariate analysis were preferentially selected for the pooled analysis for better accuracy.

### Quality assessment

The evidence level of the studies was estimated by the UK Cochrane Centre of Evidence (2009). Newcastle-Ottawa Scale [12] was utilized to assess the quality of the retrospective cohort studies. The selection of patients, comparability of the study groups, and assessment of outcome represent the critical factors of this scale, with the maximum total score of 9. Studies with scores ≥6 were defined as high-quality studies, and this was a presetting selection criterion in this report.

### Statistical analysis

The HRs and associated 95% CIs were calculated to pool the functional outcomes. Statistical heterogeneity among the studies was assessed using chi-square tests with the significance set to *P* < 0.05 or I^2^ > 50%. A fixed-effects model was utilized if there was no evident heterogeneity; otherwise, we selected a random-effects model to minimize the heterogeneity, followed by subgroup and sensitivity analysis. Funnel plots, Egger’s, and Begg’s tests were used to examine publication bias. All statistical analyses were performed using STATA version 14.0 (Stata statistical software, College Station, TX, USA).

## Results

### Characteristics of included studies

**Fig. 1:**
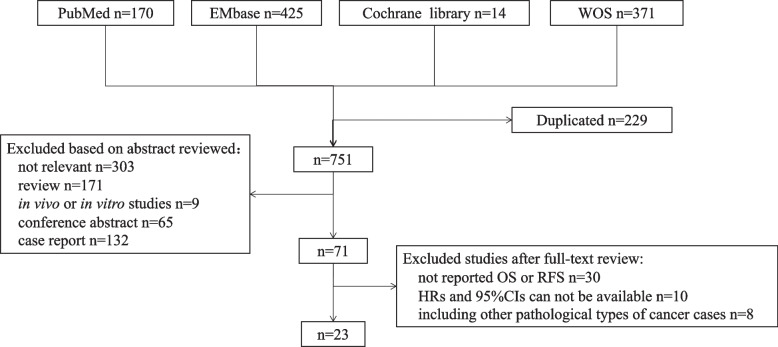
Flow of studies selection

After removing duplicated articles (*n* = 229), 751 studies were identified for review and screening. We excluded 303 unrelated studies, 171 reviews, 65 conference abstracts, 132 case reports, and 9 in vivo or in vitro studies by reading their titles and abstracts. According to the above criteria, 48 studies were excluded after full-text review due to the following reasons: 1) lack of data on OS or DFS (*n* = 30); 2) including non-biliary tract original cancers (*n* = 8); lack of HR and relevant 95%CIs (*n* = 10) (Fig. 1).

Totally, this meta-analysis enrolled 23 retrospective cohort studies with 1478 patients with BTC patients, which contains intrahepatic cholangiocarcinoma (ICC), extrahepatic cholangiocarcinoma (ECC), as well as gallbladder and ampullary cancer [8–11, 13–31]. The level of evidence is 2a. Based on the Newcastle–Ottawa Scale, all studies received a quality score of 6–9. The interested metabolic parameters of 18F-PET/CT included primary tumor maximum standardized uptake value (SUVmax) (*n* = 19), total lesion glycolysis (TLG) (*n* = 5), metabolic tumor volume (MTV) (*n* = 5), lymph nodes SUVmax (*n* = 2), and change of SUVmax (ΔSUVmax) (*n* = 3). (Table 1).

**Table 1:** Characteristics of included studies (*n* = 23)

Author, year	Country	Mean age	n (male%)	Tumor location	Parameters (cut-off)	Treatment options	follow-up time (month)	Analysis variate	Outcomes	NOS
Cho, 2015 [13]	Korea	61.0	106 (70.8%)	BTC	SUVmax (7.5)	palliative chemotherapy	7.8	M	OS	8
Furukawa, 2009 [14]	Japan	69.0	69 (58.0%)	BTC	SUVmax (6.3)	surgery, palliative therapy	53.0	U	OS	8
Harimoto, 2019 [15]	Japan	–	24 (66.7%)	ICC	SUVmax(9.6),TLG(352.8), MTV(81.2)	surgery	–	M	OS, DFS	7
Haug, 2011 [16]	Germany	–	26 (58.0%)	ICC	△SUVmax	90Y microspheres	–	U	OS	6
Hwang, 2021 [17]	Korea	68.5	55 (49.0%)	CCA	SUVmax (7.2)	chemotherapy	33.0	U	OS	7
Kim, 2019 [18]	Korea	72.0	234 (52.6%)	ECC	SUVmax (5.0), LN SUVmax (5.0)	surgery, palliative therapy	13.5	M	OS	9
Kitamura, 2010	Japan	66.0	73 (63.0%)	ECC	SUVmax (5.7)	surgery, chemotherapy	–	M	OS	7
Kobayashi, 2010	Japan	64.0	36 (na)	BTC	SUVmax (2.8)	surgery	17.2	M	OS	8
Kubo, 2022 [11]	Japan	68.0	67 (59.7%)	BTC	SUVmax (4.1), LN SUVmax (2.8)	surgery	24.0	M	OS, DFS	8
Lee, 2013 [21]	Korea	68.5	61 (52.5%)	BTC	SUVmax (5.5)	surgery, palliative therapy	–	M	OS	7
Lee, 2015 []	Korea	67.0	25 (88.0%)	ECC	SUVmax (4.0), TLG (13), MTV(2.8)	surgery	38.9	M	OS, DFS	8
Lee, 2017 [22]	Korea	68.0	76 (75.0%)	ICC	SUVmax (7.3), TLG (336.6), MTV (263.6)	surgery, palliative therapy, no therapy	5.4	M/U	OS	7
Levillain, 2019 [23]	Belgium	58.0	37 (40%)	ICC	SUVmax(9),TLG(249), MTV(59)	90Y microspheres	6.3	U	OS	8
Pak, 2014 [24]	Korea	73.0	64 (60.9%)	CCA	SUVmax(6.9)	palliative treatment	–	U	OS	6
Park,2014 [25]	Korea	61.3	64 (65.5%)	BTC	SUVmax (5.0)	surgery	27.0	M	DFS	8
Sabaté-Llobera, 2018 [26]	Spain	68.0	60 (60.0%)	CCA	SUVmax (6.6)	surgery, palliative therapy	18.5	U	OS	6
Seo,2008 [27]	Japan	64.0	27 (55.6%)	ICC	SUVmax (8.5)	surgery	20.9	M	DFS	8
Seo,2019 [8]	Japan	68.0	94 (58.8%)	ICC	SUVmax (8.0)	surgery	36.0	M	OS, DFS	9
Yi, 2018 [28]	Korea	68.0	40 (60.0%)	ICC	SUVmax (2.7), TLG (2.0), MTV (0.6)	surgery	63.3	M/U	OS	7
Yho, 2019	Japan	69.5	82 (65.1%)	ICC	SUVmax (8.0)	surgery	28.2	M	OS, DFS	9
Choi, 2017	USA	61.0	48 (70.8%)	BTC	SUVmax (8.7)	Gem±erlotinib	40.0	U	OS	7
Jo,2017 [30]	Korea	64.0	75 (57.3%)	BTC	SUVmax (9.0), △SUVmax	chemotherapy	6.8	M	OS, PFS	9
Zhu, 2010 [31]	USA	60.0	35 (60.0%)	BTC	△SUVmax	Bev + Gem+OXA	12.7	M	OS, PFS	8

*BTC* biliary track cancer, *CCA* cholangiocarcinoma, *ECC* extrahepatic cholangiocarcinoma, *ICC* intrahepatic cholangiocarcinoma, *SUVmax* maximum standardized uptake value, *△SUVmax* changed SUVmax, *LN* lymph node, *TLG* total lesion glycolysis, *MTV* metabolic tumor volume, *M* multivariate analysis, *U* univariate analysis, *OS* overall survival, *DFS* disease-free survival, *Gem* gemcitabine, *Bev* bevacizumab, *OXA* oxaliplatin

### Prognostic role of primary tumor SUVmax in OS and DFS

**Fig. 2:**
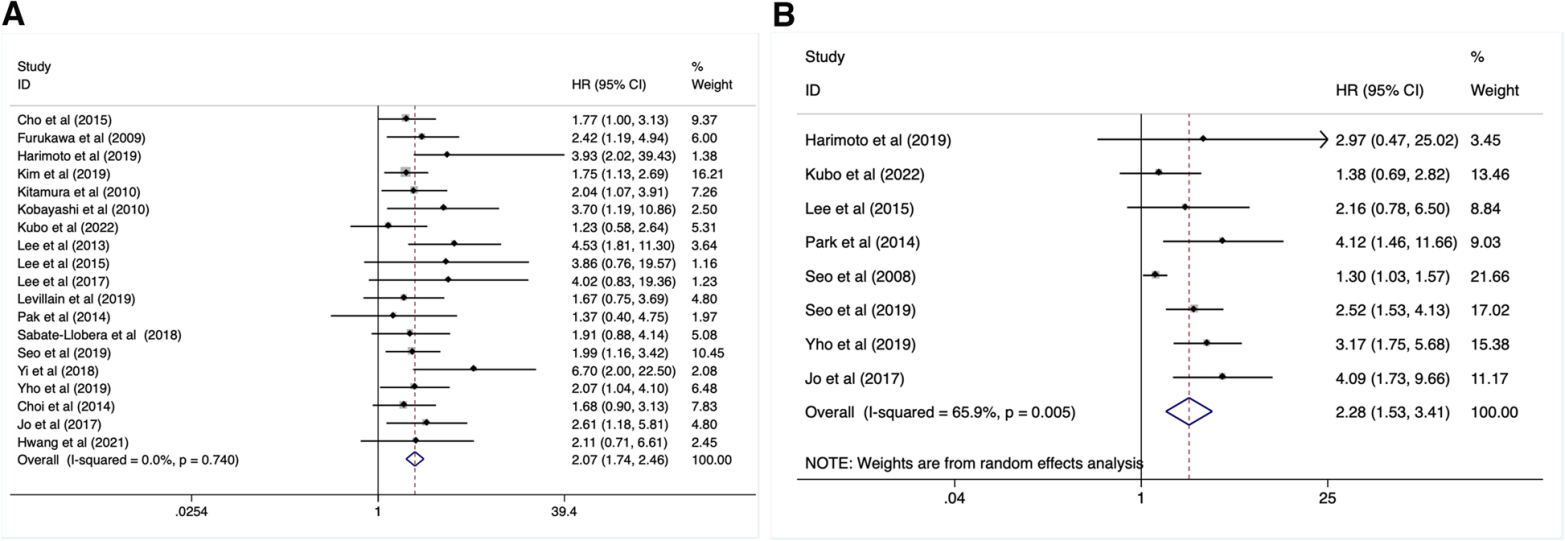
High SUVmax value indictade a poor OS (**A**) and DFS (**B**)

Nineteen studies, including 1326 patients, described the relationship between SUVmax of primary tumor and OS. Based on the result of heterogeneity (I^2^ = 13.8%, *P* = 0.740), a fixed-effect model was carried out for analysis of these data. It was revealed that an increased SUVmax was significantly associated with a worse OS (HR:2.07, 95%CI: 1.74–2.46, *P* = 0.000) (Fig. 2A, Table 2). Similarly, a higher SUVmax contributed obviously to a worse DFS (HR: 2.28, 95%CI: 1.53–3.41, *P* = 0.000) after a pooled analysis of 8 studies with 458 patients using a random-effect model (I^2^ = 69.5%, *P* = 0.005) (Fig. 2B, Table 2).

**Table 2:** Results of meta-analysis of interested outcomes

Outcomes	Cohort count	Case count	HR (95%CI)-Model	*P*	Heterogeneity		Public bias	
					I^2^ (%)	*P*-value	Begg test P	Egger test P
OS								
Tumor SUVmax	19	1326	2.07 (1.74–2.46)-fixed	0.000	13.8	0.740	0.400	0.059
TLG	5	202	1.91 (1.26–2.90)-fixed	0.002	0.0	0.484	0.142	0.083
MTV	5	202	2.04 (1.26–3.31)-fixed	0.004	8.6	0.357	0.051	0.257
ΔSUVmax	3	136	1.26 (1.06–1.50)-fixed	0.008	26.6	0.256	0.940	0.602
LN SUVmax	2	301	1.49 (0.83–2.68)-fixed	0.178	0.0	0.643	0.317	–
DFS								
Tumor SUVmax	8	458	2.28 (1.53–3.41)-random	0.000	69.5	0.005	0.881	0.068
TLG	2	49	4.34(1.42–13.27)-fixed	0.010	0.0	0.660	0.317	–
MTV	2	49	3.88(1.25–12.09)-random	0.019	52.1	0.148	0.317	–
PFS								
ΔSUVmax	2	110	1.96(0.82–4.72)-random	0.280	80.5	0.024	0.317	–

### The predictive value of TLG in OS and DFS

**Fig. 3:**
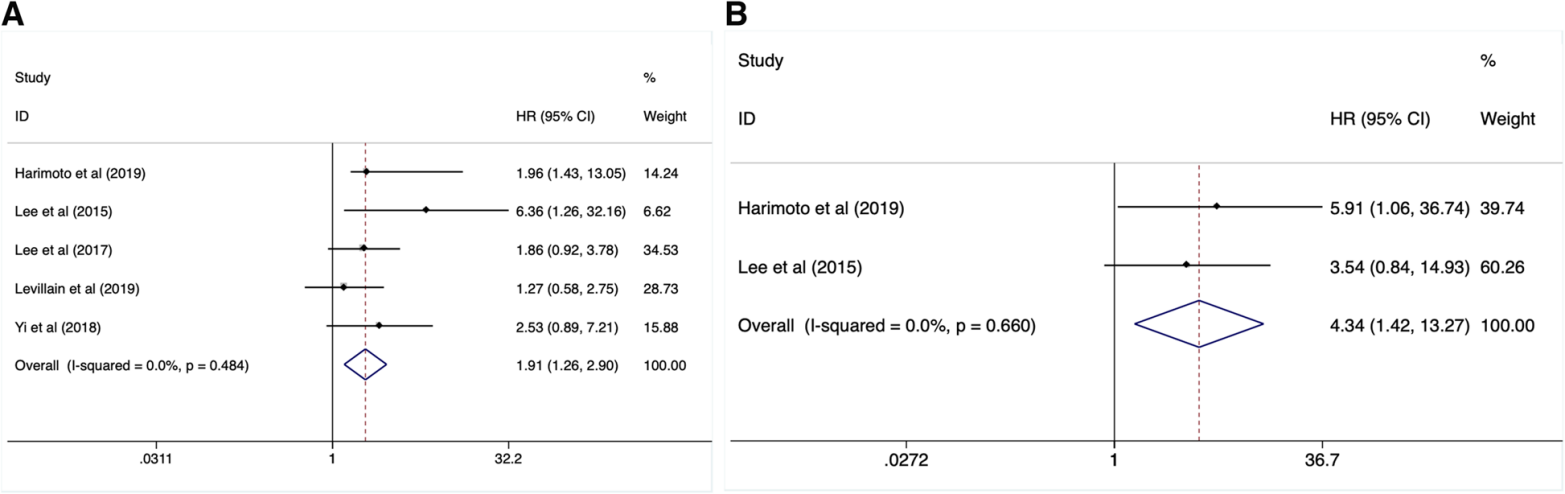
Increased TLG level predicted high risk of mortality (**A**) and disease relapse (**B**)

There were 5 studies (including 202 cases) focused on the correction of TLG and OS. After a pooled analysis with a fixed-effect model (I^2^ = 0.0%, *P* = 0.484), we found that an increased TLG linked to a higher risk of mortality (HR:1.91, 95%CI: 1.26–2.90, *P* = 0.002) (Fig. 3A, Table 2). Additionally, 2 studies with 49 patients reported the relationship between TLG and disease recurrence risk. When analyzed with a fixed-effect model (I^2^ = 0.0%, *P* = 0.660), it revealed that an increased TLG was obviously associated with poor DFS (HR: 4.34, 95%CI: 1.42–13.27, *P* = 0.01) (Fig. 3B, Table 2).

### Prognostic significance of MTV in OS and DFS

**Fig. 4:**
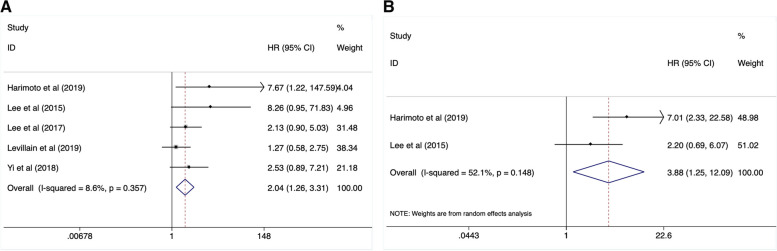
High MTV value contributed to poor OS (**A**) and DFS (**B**)

Totally, 5 studies involving 202 cases analyzed the relationship between MTV and patients` survival. As the result of pooled analysis with a fixed-effect model (I^2^ = 8.6%, *P* = 0.357), it was confirmed that an elevated MTV was significantly associated with worse OS (HR:2.04, 95%CI:1.26–3.31, *P* = 0.004) (Fig. 4A, Table 2). In addition, we demonstrated that a lower MTV contributed to better DFS significantly (HR: 3.88, 95%CI:1.25–12.09, *P* = 0.019) when analyzing 2 studies (including 49 cases) using a random-effect model (I^2^ = 52.1%, *P* = 0.148) (Fig. 4B, Table 2).

### Prognostic significance of ΔSUVmax and lymph nodes SUVmax in OS and PFS

**Fig. 5:**
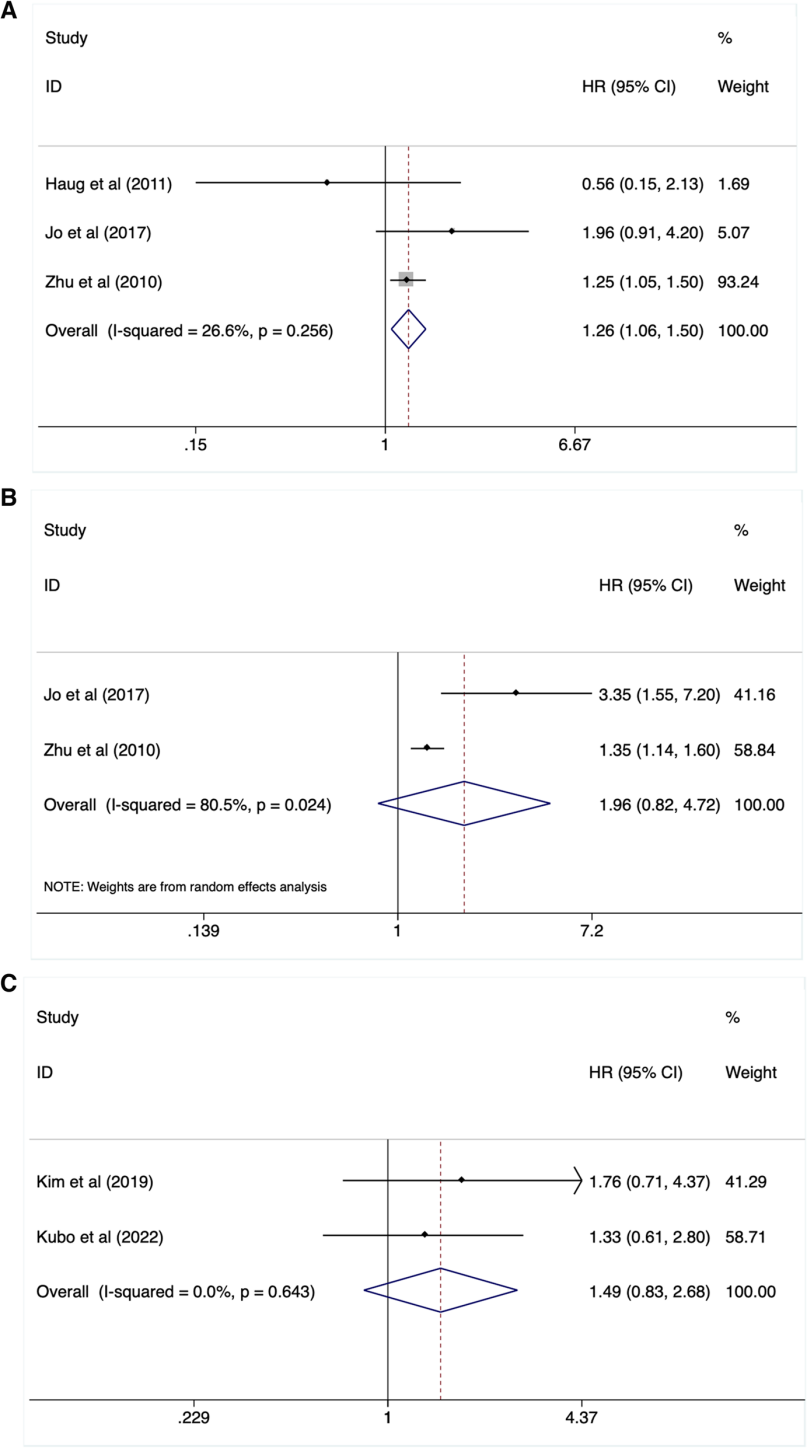
Elevated ΔSUVmax reflected worse OS (**A**) instead of DFS (**B**) Lymph node SUVmax could not predicted patients` survival (**C**)

Three studies with 136 patients reported the correction of ΔSUVmax and patients` survival. The ΔSUVmax were defined as the changes of SUVmax between pre- and post-treatment. The intervals were about 42 days, 2 months, and 3 months, respectively. After a pooled analysis with a fixed-effect model (I^2^ = 26.6%, *P* = 0.256), it demonstrated that an elevated ΔSUVmax was significantly associated with the mortality risk (HR:1.26, 95%CI:1.06–1.50, *P* = 0.008) (Fig. 5A, Table 2). 2 studies with 110 patients reported the relationship between ΔSUVmax and cancer progression risk. When analyzed with a random-effect model, we found that an increased ΔSUVmax was not associated with PFS (HR: 1.96, 95%CI: 0.82–4.72, *P* = 0.280) (Fig. 5B, Table 2).

In contrast with primary tumor SUVmax, we found that lymph nodes SUVmax (LN SUVmax) did not contribute to OS (HR: 1.49, 95%CI: 0.83–2.68, *P* = 0.178) after a pooled analysis on 2 studies (involving 301 cases) with a fixed-effect model (Fig. 5C, Table 2).

### Publication bias

**Fig. 6:**
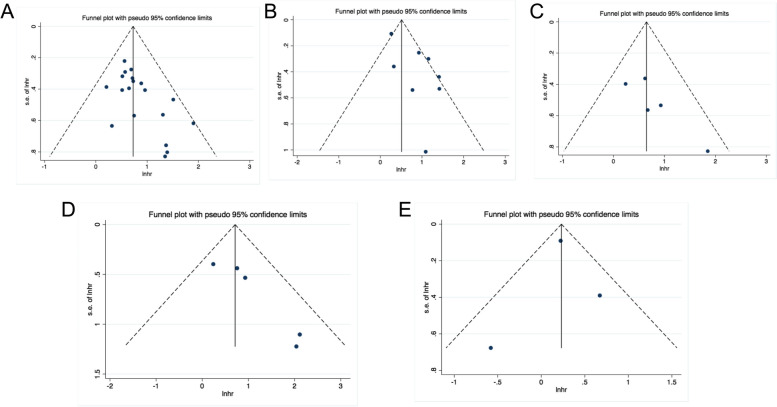
No publication bias were detected by funnel plots

Publication bias was examined by Begg’s and Egger’s tests, as well as funnel plots. All *P*-values obtained from Egger’s and Begg’s tests for each parameter and endpoint were > 0.05 (Table 2). Additionally, the visual inspection of the funnel plots did not show pronounced asymmetry (Fig. 6). These results confirmed the absence of publication bias risk among the included studies in the present meta-analysis.

## Discussion

Due to different origin sites and cancer biology, the outcomes of BTC patients are heterogeneous [32]. Therefore, the identification of reliable prognostic factors is crucial in an era of precision medicine and helps to understand the risk of disease progression and patients` mortality. The clinical-pathological features, including tumor staging information and demographic factors remain the critical consideration for clinical practice and prognosis. Interestingly, the metabolic parameters of 18F FDG PET may provide important biological information beyond the clinical-pathological characteristics in patients with BTC. Therefore, the predictive role of these parameters must be determined.

The diagnostic and staging significance of 18F FDG PET have been determined by previous meta-analyses [33–35]. However, few meta-analysis assessed and reviewed the prognostic value of this novel imaging tool. According to available studies, we performed the present meta-analysis and demonstrated that higher values of SUVmax, MTV, and TLG predicted a higher risk of disease recurrence or death in patients with BTC. In addition, the emerging parameters such as LN SUVmax and ΔSUVmax have also been focused by our study, but the prognostic value needs further investigation due to insufficient published studies. These findings suggest that 18F FDG PET is not only a diagnostic tool but may be used to distinguish BTC patients who are at high risk of tumor recurrence or death and may benefit from subsequent, more aggressive treatments.

SUVmax is the most commonly used parameter in 18F FDG PET diagnosis and response monitoring because of its high reproducibility and availability. FDG uptake can reflect the metabolic activity of the tumor tissue. SUVmax has been shown to correlate with tumor mitotic count and with prognosis in cancer patients [36]. Previous studies demonstrated that the value of SUVmax was associated with multiple clinical-pathological features, including histological grade [37]. In addition, it has been uncovered that SUVmax was significantly correlated with programmed cell death ligand 1 (PD-L1) (*P* = 0.02) and glucose transporter 1 (GLUT1) (*P* < 0.01) expression in patients with pulmonary squamous-cell carcinoma [38]. Besides, SUVmax has been revealed as a marker associated with low tumor-infiltrating lymphocyte levels [39]. These results suggested that SUVmax, as a prognostic factor, could reflect cancer immune microenvironment. Interestingly, SUVmax was also associated with tumor tissue hypoxia and angiogenesis, contributing to cancer progression [40, 41].

MTV and TLG, which are a combination of volumetric and metabolic parameters, may be utilized in metabolic analyses of radiotracer activity, reflecting both properties of the tumor tissues. These parameters were also related to angiogenesis [41] and cancer immunity [42]. Taken these considerations, the above parameters in 18F PDF PET were associated to glucose intake, which regards cancer metabolism activity, tumor microenvironment, and immunity. These biological features contribute to tumor growth, cancer relapse, treatment resistance, and metastasis. Thereby, 18F PDF PET could be considered as a prognostic tool in patients with cancer including BTC.

previous studies demonstrated that glycolysis, as metabolic reprogramming, contributed significantly to cholangiocarcinoma initiation and progression [43, 44] Glycolic pathways and enzymes, including pyruvate kinase M2 [45, 46], Aldolase A [47] and lactate dehydrogenase A [48], play a critical role in BTC and have been utilized as biomarkers to predict patients` outcomes. Targeting glycolysis could be considered as a promising treatment option in BTC [49–51].

Nevertheless, some limitations in the present study should be acknowledged. Firstly, all included investigations were retrospective cohort studies with a small sample size and a modest level of evidence. Moreover, most participants in these studies were from Asian countries (e.g., Korea and Japan), which may be restricted to other regions. In addition, the cut-off value of interested parameters in each study was inconsistent, which needs further exploration of standard and optimal values for clinical practice. Besides, the included studies about some parameters (i.e., TLG, MTV,ΔSUVmax and LN SUVmax) were insufficient. Lastly, several HRs and their 95% CIs were extracted from univariate analysis, which might lead to an overestimation of the prognostic value of these markers.

## Conclusion

In conclusion, this study demonstrated that 18F FDG PET parameters are associated with the risk of death. Especially SUVmax, TLG, MTV, and ΔSUVmax perform well in BTC patients` future survival analysis. Despite some limitations, we confirmed that 18F FDG PET could be a valuable method to help predict survival outcomes in biliary cancer patients.

### Supplementary Information

**Additional file 1.**

## Data Availability

The datasets used and/or analyzed during the current study are available from the corresponding author on reasonable request.

## Abbreviations

18F FDG PET: 18F-fluorodeoxyglucose positron-emission tomography
TLG: Total lesion glycolysis
MTV: Metabolic tumor volume
SUVmax: Maximum Standardized uptake value
ΔSUVmax: Change of maximum Standardized uptake value
BTC: Biliary tract cancer
ICC: Intrahepatic cholangiocarcinoma
ECC: Extrahepatic cholangiocarcinoma
LN: Lymph node
OS: Overall survival
DFS: Disease-free survival
PFS: Progression-free survival
HR: Hazard ratio
CIs: Confidence intervals
PD-L1: Programmed cell death ligand 1
GLUT1: Glucose transporter 1
